# Effect of supplementation with *yeast polysaccharides* on intestinal function in piglets infected with porcine epidemic diarrhea virus

**DOI:** 10.3389/fmicb.2024.1378070

**Published:** 2024-04-09

**Authors:** Hanxiao Li, Mengjun Wu, Zhonghua Li, Qian Zhang, Xiaohan Zhang, Yanyan Zhang, Di Zhao, Lei Wang, Yongqing Hou, Tao Wu

**Affiliations:** Hubei Key Laboratory of Animal Nutrition and Feed Science, Wuhan Polytechnic University, Wuhan, China

**Keywords:** piglets, porcine epidemic diarrhea virus, intestine function, *yeast polysaccharides*, nutrition

## Abstract

Porcine epidemic diarrhea virus (PEDV) has caused huge economic losses to the pig industry. Yeast polysaccharides (YP) has been used as a feed additive in recent years and poses good anti-inflammatory and antiviral effects. The present study aimed to explore the protective effect of YP on intestinal damage in PEDV-infected piglets. Eighteen 7-day-old piglets with similar body weights were randomly divided into three groups: Control group (basal diet), PEDV group (basal diet), and PEDV+YP group (basal diet +20 mg/kg BW YP), six replicates per group and one pig per replicate. Piglets in PEDV group and PEDV+YP group were orally given PEDV (dose: 1 × 10^6^ TCID_50_) at 19:30 PM on the 8th day of the experiment. The control group received the same volume of PBS solution. Weight was taken on an empty stomach in the morning of the 11th day, blood was collected and then anesthetic was administered with pentobarbital sodium (50 mg/kg·BW) by intramuscular injection, and samples were slaughtered after the anesthetic was complete. The results showed that YP could alleviate the destruction of intestinal villus morphology of piglets caused by PEDV. Meanwhile, PEDV infection can reduce the activity of glutathione peroxidase, superoxide dismutase and catalase, and increase the content of malondialdehyde. YP can improve the antioxidative capacity in the serum and small intestine of PEDV-infected piglets. In addition, YP inhibited the replication of PEDV in the jejunum ileum and colon. Moreover, YP can regulate the mRNA levels of inflammatory genes (IL-1β and iNOS) and lipid metabolic genes (APOA4 and APOC3) in the small intestine. In summary, YP could inhibit virus replicates, improve intestinal morphology, enhance antioxidant capacity, relieve inflammation and regulate the metabolism of the intestine in PEDV-infected piglets.

## 1 Introduction

Porcine epidemic diarrhea virus (PEDV) is a virus belonging to the genus alpha coronavirus of the coronaviruses family, which can cause acute diarrhea/vomiting, dehydration and high mortality of newborn piglets (Jung and Saif, 2015). Infection with PEDV in newborn piglets results in fecal virus shedding (PEDV RNA can be frequently detected in the nasal cavity), acute toxemia, severe atrophic enteritis (mainly jejunum and ileum), and increased pro-inflammatory and congenital immune responses (Jung et al., 2020). Clinical practice has shown that even vaccinated pigs do not protect against PEDV infection, indicating that the vaccine derived from the classical strain has insufficient protection, a phenomenon that may be caused by the sharp mutation of the virus, which poses a major challenge for PED prevention and control in China (Wang et al., 2016). Until now, safe and effective drugs and feed additives are unavailable.

Yeast polysaccharides (YP), a new bioactive compound, is mainly composed of β-Glucans and mannan, has attracted attention because of its efficient detoxification and non-polluting effect. It is known that YP reveal immunomodulating properties, which allows for their application in anti-infective and antitumor therapy (Kogan et al., 2008). Recent data also suggest that polysaccharides reveal antioxidant activity that can result in their protective function as antioxidants, antimutagens, and antigenotoxic agents (Castillo et al., 2008). Study shows that yeast β-glucan and milk hydrolysate is a suitable alternative to zinc oxide in the race to alleviate post-weaning diarrhea in piglets (Mukhopadhya et al., 2019). In recent years, more and more studies have been conducted on the application of YP in pig diets. Liu Y et al. ‘s study shows that maternal dietary supplementation of yeast cultures improved the immune performance of weaned piglets by inhibiting inflammatory responses (Liu et al., 2023). The result of Zhao Y et al. showed that biomacromolecules mannan/β-glucans from yeast cell wall can improve cell morphology and activity, weaken oxidative damage, and reduce autophagy induced by deoxynivalenol (Zhao et al., 2020). But there are only few reports about the effect of YP on PEDV in piglets. Therefore, the present study aimed to explore the protective effect of YP on intestinal damage in PEDV-infected piglets, providing the new insight of developing green feed additive to prevent PED.

## 2 Materials and methods

### 2.1 Animal experiments

Single factor design was employed to study the protective effect of YP on intestinal tract of PEDV-infected piglets. 18 7-day-old healthy piglets with an average body weight of 2.5 ± 0.3 kg were randomly divided into 3 groups: Control group, PEDV group, and PEDV+ YP group with 6 replicates per group. The total experimental period was 11 days, the adaptation period was 3 days, and the formal experimental period was 8 days, during which all groups were fed the same experimental diet. On days 4–10 of the experiment, piglets in PEDV+ YP group were given oral administration of YP (made of artificial milk suspension with dosage of 20 mg/kg BW) at 19:30 every night, and piglets in Control group and PEDV group were given oral administration of artificial milk of equal volume. (The nutritional levels of the milk replacer is shown in Table 1.) Piglets in PEDV group and PEDV+YP group were orally given PEDV (dose: 1 × 10^6^ TCID_50_) at 19:30 PM on the 8th day of the experiment. Water and feed were cut off at 22:00 on the 10th day of the experiment, and weight was taken on an empty stomach in the morning of the 11th day, blood was collected and then anesthetic was administered with pentobarbital sodium (50 mg/kg·BW) by intramuscular injection, and samples were slaughtered after the anesthetic was complete. The dose of YP used in this study was determined based on pretest, all procedures were approved by the Animal Care and Use Committee of Wuhan Polytechnic University (Index number: WPU202209004).

**Table 1:** Nutritional levels of the milk replacer (%).

Items	Crude protein	Crude fat	Crude ash	Crude fiber	Water	Lysine	Calcium	Total phosphorus
Milk replacer	≥20.0	≥10.0	≥9.0	≥0.3	≤10.0	≥1.4	0.4 ~ 1.1	≥0.3

### 2.2 Intestinal morphology

One centimeter long small intestine sample were fixed with 4% paraformaldehyde. The fixed samples are then dehydrated and embedded with paraffin wax. Sections with a thickness of 6 μm were dewaxed with xylene and stained with hematoxylin and eosin (H&E). Images of pathological sections of the intestine were obtained using a DM3000 microscope (Leica Microsystems, Wetzlar, Germany). Ten complete intestinal villi were randomly selected for measurement. Olympus BX41 microscope (Olympus, Tokyo, Japan) and imagepro Plus 6.0 software (Media Cybernetics, Rockville, Japan MD) measured villus height (VH), crypt depth (CD), villus area (VA), and villus height/crypt depth (VH/CD).

### 2.3 Blood sample collection and blood biochemical measurements in plasma

On the morning of the 11th day of the experiment, blood was collected from the anterior vena cava of piglets using EDTA anticoagulant blood collection vessel, common vacuum blood collection vessel and disposable blood collection needle, and the blood collection vessel was gently shaken to prevent blood coagulation. After blood collection, the collection vessel is placed on ice, and the plasma and serum are separated in time. Then, the blood sample is stored in the refrigerator at −80°C for the convenience of subsequent detection. Concentrations of biochemical parameters in plasma (TB, TP, ALB, ALT, ALP, TC, TG, GLU, CA, CREA, BUN, GGT, CK, DB, LDH) were measured with corresponding kits using a Hitachi 7,060 Automatic Biochemical Analyzer (Hitachi, Japan).

### 2.4 Activities of anti-oxidant enzymes and concentrations of oxidation-relevant products in serum and intestinal tract mucosa

Serum and intestinal tract mucosae were used for the analysis of anti-oxidative enzymes and related products. The activity and content of superoxide dismutase (SOD), glutathione peroxidase (GSH-Px), catalase (CAT), myeloperoxidase (MPO), malondialdehyde (MDA) and hydrogen peroxide (H_2_O_2_) were detected by kit (Nanjing Institute of Jiancheng Bioengineering Institute, Nanjing, China). Follow the kit instructions strictly (Table 1).

### 2.5 Detection of mRNA expression levels by real-time quantitative PCR

Total RNA was extracted from small intestine with RNAiso Plus (Takara, Dalian, China) reagent. The cDNA was then synthesized using PrimeScript^®^RT kit with gDNA Eraser (Takara, Dalian, China). At last, real-time quantitative PCR was performed using SYBR^®^Premix Ex Taq™(Tli RNaseHPlus) (Takara, Dalian, China). Gene expression was determined using the 2-DDCt method relative to the values in control group after normalization to housekeeping genes RPL19. The primer sequences used for this study were listed below (Table 2). The mRNA expression levels detected in this study included viral replication genes (PEDV M, PEDV N, PEDV S), intestinal tissue damage related genes (iFABP, AREG, MMP13), intestinal inflammatory response genes (IL-1β, REG3G, IRF7), lipid metabolism genes (APOA4, APOC3), ion channel genes (NHE3, TRPV6) and antioxidant genes (iNOS, GSTO2).

**Table 2:** Sequence of the primers used for qPCR analysis.

Gene	Forward (5′ -3′)	Reverse (5′ -3′)
RPL19	AACTCCCGTCAGCAGATCC	AGTACCCTTCCGCTTACCG
PEDV M	TCCCGTTGATGAGGTGAT	AGGATGCTGAAAGCGAAAA
PEDV N	TTGGTGGTAATGTGGCTGTTC	TGGTTTCACGCTTGTTCTTCTT
PEDV S	CTCTCTGGTACAGGCAGCAC	GCTCACGTAGAGTCAAGGCA
IFABP	GAAACTTGCAGCTCATGACAAT	GTCTGCGAGGCTGTAGTTAAA
AREG	GAGTACGATAACGAACCGCACA	TTTCCACTTTTGCCTCCCTTT
MMP13	AGTTTGGCCATTCCTTAGGTCTTG	GGCTTTTGCCAGTGTAGGTATAGAT
IL-1β	CAACGTGCAGTCTATGGAGT	GAGGTGCTGATGTACCAGTTG
REG3G	CTGTCTCAGGTCCAAGGTGAAG	CAAGGCATAGCAGTAGGAAGCA
IRF7	CAGAAGCAGCTCCACTACAC	CTCCCAGTAGACTTTGCACTT
INOS	CTCCAGGTGCCCACGGGAAA	TGGGGATACACTCGCCCGCC
GSTO2	GCCTTGAGATGTGGGAGAGAA	AAGATGGTGTTCTGATAGCCAAGA
NHE3	AAGTACGTGAAGGCCAACATCTC	TTCTCCTTGACCTTGTTCTCGTC
TRPV6	AGGAGCTGGTGAGCCTCAAGT	GGGGTCAGTTTGGTTGTTGG
APOA4	ACCCAGCAGCTCAACACTCTC	GAGTCCTTGGTCAGGCGTTC
APOC3	CTAACCAGCGTGAAGGAGTC	CAGAAGTCGGTGAACTTGCC

Values are mean and pooled SEM, *n* = 6; RPL19: L19 ribosomal protein gene; PEDV M: membrane protein (porcine epidemic diarrhea virus); PEDV N: nucleocapsid protein (porcine epidemic diarrhea virus); PEDV S: spike protein (porcine epidemic diarrhea virus); IFABP: intestinal fatty acid binding protein; AREG: amphiregulin; MMP13: matrix metalloproteinase-13; IL-1β: nterleukin-1 beta; REG3G: regenerating islet-derived 3 gamma; IRF7: interferon regulatory factor 7; INOS: inducible nitric oxide synthase; GSTO2: glutathione-s-transferase omega 2; NHE3:Na(+)-H(+) exchanger 3; TRPV6: transient receptor potential vanilloid 6; APOA4: apolipoprotein A-IV; APOC3: apolipoprotein C3; a,b,c within a row, means with different superscripts differ, *p* < 0.05.

### 2.6 Statistical analysis

The experimental data were sorted by Excel software, and then the comparative mean model (single factor ANOVA test) in statistical software SPSS 25.0 was used to conduct one-way ANOVA and Duncan’s multiple comparisons. *p* < 0.05 indicates a significant difference, *p* < 0.1 indicates a significant trend of difference, and the test results are expressed by mean value and standard error (SEM).

## 3 Results

### 3.1 Intestinal morphology

**Figure. 1:**
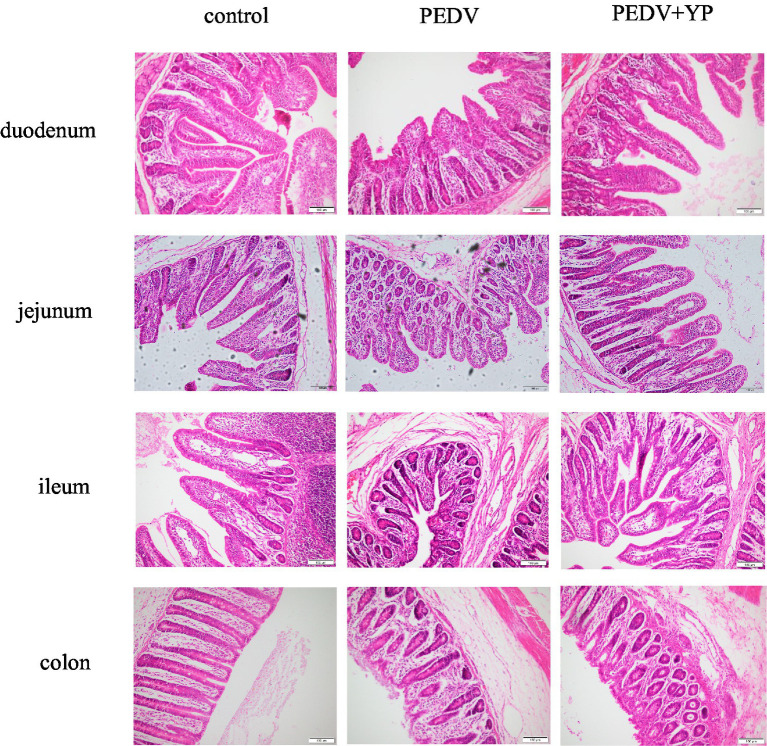
Histopathological structures of piglet’s duodenum, jejunum, ileum and colon in the Control, PEDV and PEDV + YP groups.

Data on the small intestinal morphology are summarized in Table 3. Compared with the control group, PEDV infection decreased the VH and VA of duodenum, jejunum and ileum, and reduced VH/CD of duodenum and jejunum (*p* < 0.05). Meanwhile, PEDV infection increased crypt depth of duodenum and colon (*p* < 0.05). Compared with the PEDV group, feeding YP significantly decreased the CD of jejunum and ileum (*p* < 0.05), the VA of duodenum and jejunum was significantly increased (*p* < 0.05), and the VH and VH/CD of duodenum were significantly increased (*p* < 0.05). As shown in Figure 1, PEDV infection caused typical PED symptoms with multifocal to diffuse villus atrophy. Obviously, piglets in the PEDV+YP group exhibited less intestinal lesions than those in the PEDV group.

**Table 3:** Effects of YP administration on intestinal morphology in piglets infected with PEDV.

Items	Control	PEDV	PEDV+YP	*p*-value
*duodenum*				
VH (μm)	373.300 ± 61.310^a^	253.676 ± 36.868^b^	358.426 ± 51.496^a^	<0.001
CD (μm)	136.234 ± 13.163^b^	156.581 ± 9.720^a^	144.076 ± 11.639^ab^	0.006
VA (μm^2^)	35661.731 ± 10687.806^a^	21352.932 ± 4858.366^b^	32490.682 ± 4887.441^a^	0.003
VH/CD	2.603 ± 0.455^a^	1.787 ± 0.277^b^	2.415 ± 0.166^a^	<0.001
*jejunum*				
VH (μm)	381.593 ± 26.221^a^	294.554 ± 44.511^b^	327.185 ± 49.752^b^	0.001
CD (μm)	111.981 ± 8.800^a^	121.375 ± 14.906^a^	95.248 ± 16.196^b^	0.009
VA (μm^2^)	34172.984 ± 6184.644^a^	25626.223 ± 5284.460^b^	32701.427 ± 3622.767^a^	0.009
VH/CD	3.313 ± 0.256^a^	2.739 ± 0.377^b^	3.187 ± 0.592^ab^	0.02
*ileum*				
VH (μm)	329.953 ± 36.755^a^	261.507 ± 30.567^b^	252.916 ± 29.692^b^	<0.001
CD (μm)	101.929 ± 11.477^a^	102.149 ± 6.633^a^	87.837 ± 13.598^b^	0.045
VA (μm^2^)	29804.723 ± 5452.772^a^	20262.433 ± 4157.977^b^	20368.862 ± 2418.766^b^	<0.001
VH/CD	3.078 ± 0.287	2.849 ± 0.196	2.798 ± 0.247	0.093
*colon*				
CD (μm)	290.335 ± 11.802^b^	334.538 ± 35.818^a^	278.393 ± 22.769^b^	0.001

Values are mean and pooled SEM, *n* = 6; VH, villus height; CD, crypt depth; VA, villus area; a,b,c within a row, means with different superscripts differ, *p* < 0.05.

### 3.2 Plasma biochemical parameters

Data on plasma biochemical parameters are summarized in Table 4. Compared with the control group, PEDV infection decreased the content of TG, CA and GGT (*p* < 0.05), increased the content of CK (*p* < 0.05). Compared with the PEDV group, the TG, CA and GGT content increased and CK was decreased in the PEDV+YP group (*p* < 0.05).

**Table 4:** Effects of YP administration on plasma biochemistry in piglets infected with PEDV.

Items	Control	PEDV	PEDV+YP	*p-*value
TB	2.535 ± 0.575	2.275 ± 0.706	2.678 ± 0.664	0.511
TP	58.504 ± 6.500	61.563 ± 7.209	63.232 ± 4.220	0.410
ALB	29.388 ± 4.699	32.013 ± 2.203	32.762 ± 4.556	0.243
ALT	30.875 ± 6.792	32.667 ± 7.746	31.000 ± 5.831	0.850
ALP	264.400 ± 12.361	271.822 ± 39.039	288.520 ± 64.72	0.196
TC	85.252 ± 19.539	79.674 ± 14.753	105.376 ± 24.309	0.157
TG	26.997 ± 7.592^a^	9.734 ± 4.084^b^	23.553 ± 8.766^a^	<0.001
GLU	114.880 ± 28.697	115.600 ± 23.399	127.860 ± 14.165	0.656
CA	14.168 ± 0.899^a^	12.479 ± 0.309^b^	13.718 ± 0.958^a^	<0.001
CREA	0.608 ± 0.129	0.883 ± 0.342	0.864 ± 0.264	0.280
BUN	6.920 ± 1.173	10.880 ± 4.643	10.101 ± 4.233	0.231
GGT	28.084 ± 3.819^a^	21.359 ± 4.312^b^	29.520 ± 1.911^a^	<0.001
CK	183.600 ± 42.770^b^	256.467 ± 120.604^a^	185.120 ± 36.559^b^	<0.001
DB	0.114 ± 0.018	0.086 ± 0.036	0.105 ± 0.023	0.454
LDH	552.200 ± 70.889	629.540 ± 167.758	609.000 ± 83.865	0.433

Values are mean and pooled SEM, *n* = 6; TB: total bilirubin; TP: total protein; ALB: albumin; ALT: Alanine amino transferase; ALP: alkaline phosphatase; TC: total cholesterol; TG: triglyceride; GLU: glucose; Ca: Calcium; CREA: creatinine; BUN: blood urea nitrogen; GGT: glutamyltranspeptidase; CK: creatine kinase; DB: direct bilirubin; LDH: lactate dehydrogenase; a,b,c within a row, means with different superscripts differ, *p* < 0.05.

### 3.3 Activities of anti-oxidant enzymes and concentrations of oxidation-relevant products in serum and intestinal tract mucosa

Data on GSH-Px, SOD, CAT and MPO activities, MDA and H_2_O_2_ concentrations are summarized in Table 5. Compared with control group, PEDV infection decreased the activities of GSH-Px in duodenum and ileum; decreased the activities of SOD in duodenum and ileum, increased the activities of SOD in serum; decreased the activities of CAT in ileum; increased the activities of MPO in ileum and colon; increased the concentration of MDA in serum, duodenum, jejunum and colon (*p* < 0.05). Compared with the PEDV group, feeding YP increased the activities of GSH-Px in serum; increased the activities of SOD in ileum and colon; decreased the activities of CAT in jejunum, increased the activities of CAT in ileum and colon; decreased the activities of MPO in serum, ileum and colon; decreased the concentrations of MDA in serum, duodenum and jejunum; decreased the concentrations of H_2_O_2_ in serum and duodenum (*p* < 0.05).

**Table 5:** Effects of YP administration on redox status in piglets infected with PEDV.

Items	Control	PEDV	PEDV+YP	*p-*value
*GSH-Px*				
serum	330.360 ± 12.285^b^	332.669 ± 17.179^b^	375.828 ± 39.710^a^	0.004
duodenum	100.373 ± 13.526^a^	80.839 ± 8.589^b^	86.760 ± 7.183^b^	0.004
jejunum	91.443 ± 12.021	83.583 ± 10.264	98.670 ± 27.517	0.252
ileum	109.616 ± 9.100^a^	89.170 ± 12.902^b^	109.374 ± 18.514^a^	0.008
colon	69.589 ± 14.199	56.516 ± 8.635	68.002 ± 18.321	0.121
*SOD*				
serum	71.107 ± 3.049^b^	77.455 ± 3.963^a^	75.511 ± 5.360^ab^	0.014
duodenum	447.505 ± 21.861^a^	393.071 ± 38.280^b^	422.133 ± 31.855^ab^	0.008
jejunum	415.895 ± 54.986	369.666 ± 26.012	375.489 ± 46.042	0.092
ileum	297.276 ± 24.323^a^	252.184 ± 23.094^b^	281.619 ± 16.690^a^	0.002
colon	299.052 ± 16.306^ab^	260.035 ± 55.999^b^	312.441 ± 30.325^a^	0.055
*CAT*				
serum	2.586 ± 1.272	2.201 ± 0.545	2.294 ± 0.585	0.666
duodenum	7.602 ± 1.164	7.161 ± 0.484	7.020 ± 0.424	0.381
jejunum	6.380 ± 0.445^a^	5.992 ± 0.987^a^	3.613 ± 0.882^b^	<0.001
ileum	1.130 ± 0.236^b^	0.761 ± 0.138^c^	2.427 ± 0.291^a^	<0.001
colon	2.055 ± 0.320^b^	2.173 ± 0.495^b^	3.004 ± 0.594^a^	0.005
*MPO*				
serum	44.290 ± 4.067^a^	48.891 ± 3.801^a^	34.102 ± 6.701^b^	<0.001
duodenum	176.388 ± 15.618	182.718 ± 22.507	177.797 ± 4.256	0.744
jejunum	192.025 ± 26.867	193.777 ± 23.263	167.847 ± 18.486	0.142
ileum	195.988 ± 24.071^b^	242.345 ± 31.132^a^	208.358 ± 29.640^b^	0.01
colon	183.123 ± 14.253^b^	212.863 ± 23.856^a^	155.359 ± 19.465^c^	<0.001
*MDA*				
serum	1.913 ± 0.297^b^	2.481 ± 0.456^a^	1.880 ± 0.220^b^	0.006
duodenum	0.506 ± 0.092^b^	0.813 ± 0.111^a^	0.610 ± 0.158^b^	<0.001
jejunum	0.438 ± 0.064^b^	0.604 ± 0.137^a^	0.345 ± 0.087^b^	0.001
ileum	0.603 ± 0.095	0.618 ± 0.094	0.547 ± 0.036	0.344
colon	0.359 ± 0.079^b^	0.477 ± 0.091^a^	0.447 ± 0.105^ab^	0.041
*H_2_O_2_*				
serum	43.817 ± 4.311^ab^	51.678 ± 10.772^a^	37.227 ± 10.216^b^	0.024
duodenum	13.704 ± 0.765^a^	13.818 ± 1.072^a^	11.643 ± 2.346^b^	0.022
jejunum	12.994 ± 1.544	14.711 ± 3.084	12.091 ± 2.909	0.179
ileum	13.021 ± 0.953	13.435 ± 1.916	12.097 ± 0.579	0.253
colon	11.348 ± 1.497	11.910 ± 1.243	10.807 ± 0.400	0.285

Values are mean and pooled SEM, *n* = 6; CAT: catalase; MPO: myeloperoxidase; GSH-Px: glutathione peroxidase; H_2_O_2_: hydrogen peroxide; MDA: malondialdehyde; a,b,c Within a row, means with different superscripts differ, *p* < 0.05.

### 3.4 The mRNA levels of porcine epidemic diarrhea virus related gene in jejunum, ileum, and colon

**Figure. 2:**
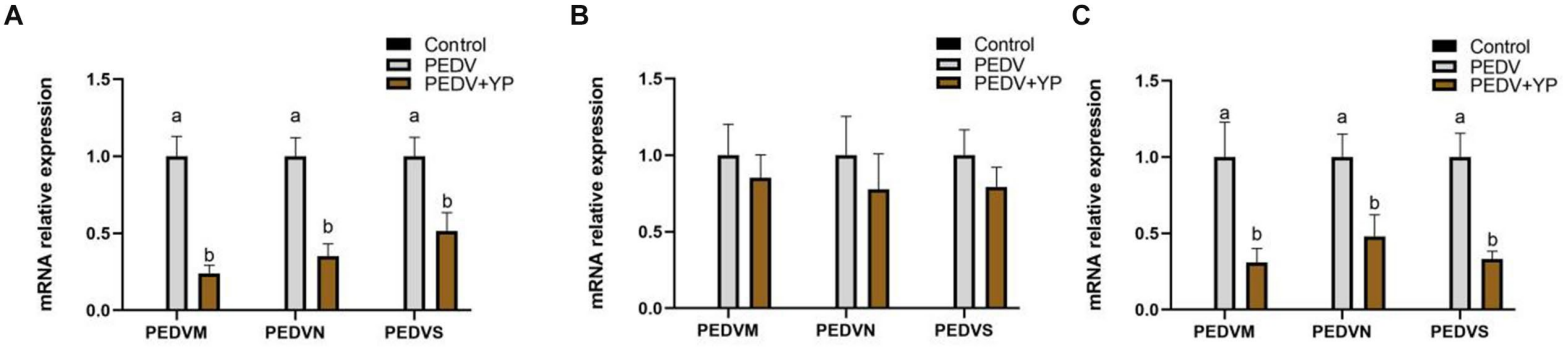
Effects of YP on the expression of mRNA expression levels of and detected by qPCR in the jejunum, ileum and colon of piglets. **(A)** jejunum; **(B)** ileum; **(C)** colon. Values are mean and pooled SEM, *n* = 6; a,b means with different superscripts differ, *p* < 0.05.

Data on PEDV M, PEDV N and PEDV S mRNA levels are summarized in Figures 2A–C (A, B and C represent the jejunum ileum and colon). Compared with the PEDV group, piglets fed by YP had lower PEDV M, PEDV N and PEDV S mRNA levels in the jejunum and colon (*p* < 0.05).

### 3.5 The mRNA levels of intestinal tissue damage related genes in jejunum, ileum, and colon

**Figure. 3:**
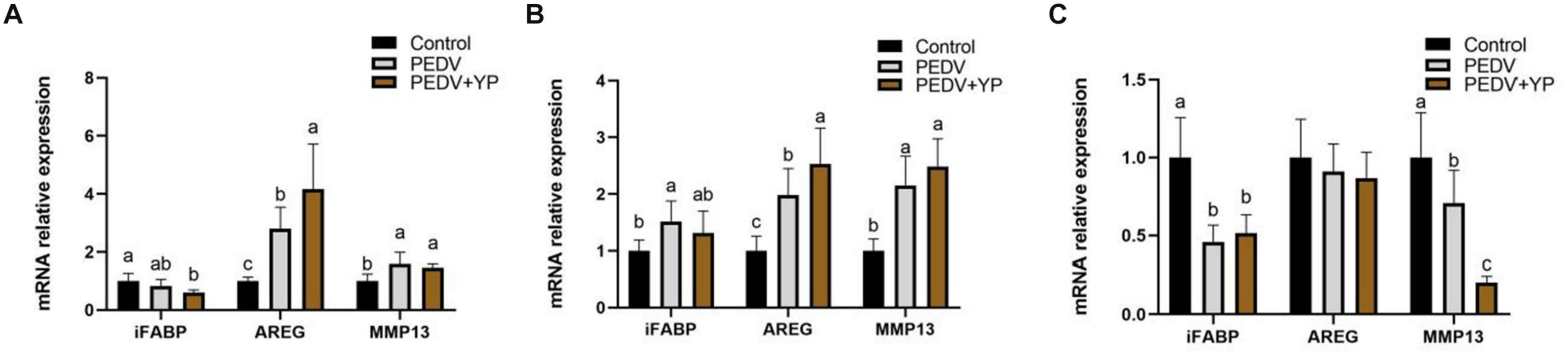
Effects of YP on the expression of mRNA expression levels of and detected by qPCR in the jejunum, ileum and colon of piglets. **(A)** jejunum; **(B)** ileum; **(C)** colon. Values are mean and pooled SEM, *n* = 6; a,b,c means with different superscripts differ, *p* < 0.05.

Data on iFABP, AREG and MMP13 mRNA levels are summarized in Figures 3A–C (A, B and C represent the jejunum ileum and colon). Compared with control group, PEDV-infected piglets had higher AREG and MMP13 mRNA levels in the jejunum; higher iFABP, AREG and MMP13 mRNA levels in the ileum; lower iFABP and MMP13 mRNA levels in the colon (*p* < 0.05). Compared with PEDV group, piglets fed by YP had a higher AREG mRNA level in the jejunum and ileum; a lower MMP13 mRNA level in the colon (*p* < 0.05).

### 3.6 The mRNA levels of genes associated with intestinal inflammatory response in jejunum, ileum, and colon

**Figure. 4:**
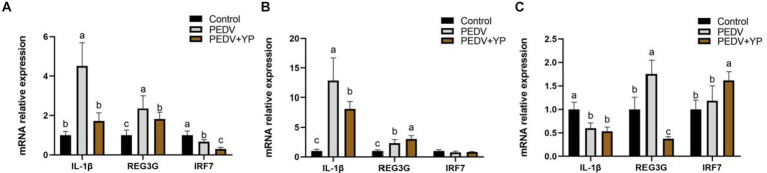
Effects of YP on the expression of mRNA expression levels of and detected by qPCR in the jejunum, ileum and colon of piglets. **(A)** jejunum; **(B)** ileum; **(C)** colon. Values are mean and pooled SEM, *n* = 6; a,b,c means with different superscripts differ, *p* < 0.05.

Data on IL-1β, REG3G and IRF7 mRNA levels are summarized in Figures 4A–C (A, B and C represent the jejunum ileum and colon). Compared with control group, PEDV-infected piglets had higher IL-1β and REG3G mRNA levels in the jejunum and ileum; a lower IRF7 mRNA level in the jejunum; a lower IL-1β but higher REG3G mRNA level in the colon (*p* < 0.05). Compared with PEDV group, piglets fed by YP had lower IL-1β, REG3G and IRF7 mRNA levels in the jejunum; a lower IL-1β but higher REG3G mRNA level in the ileum; a lower REG3G but higher IRF7 mRNA level in the colon (*p* < 0.05).

### 3.7 The mRNA levels of lipid metabolism genes, ion channel genes, and antioxidant genes in jejunum

**Figure. 5:**
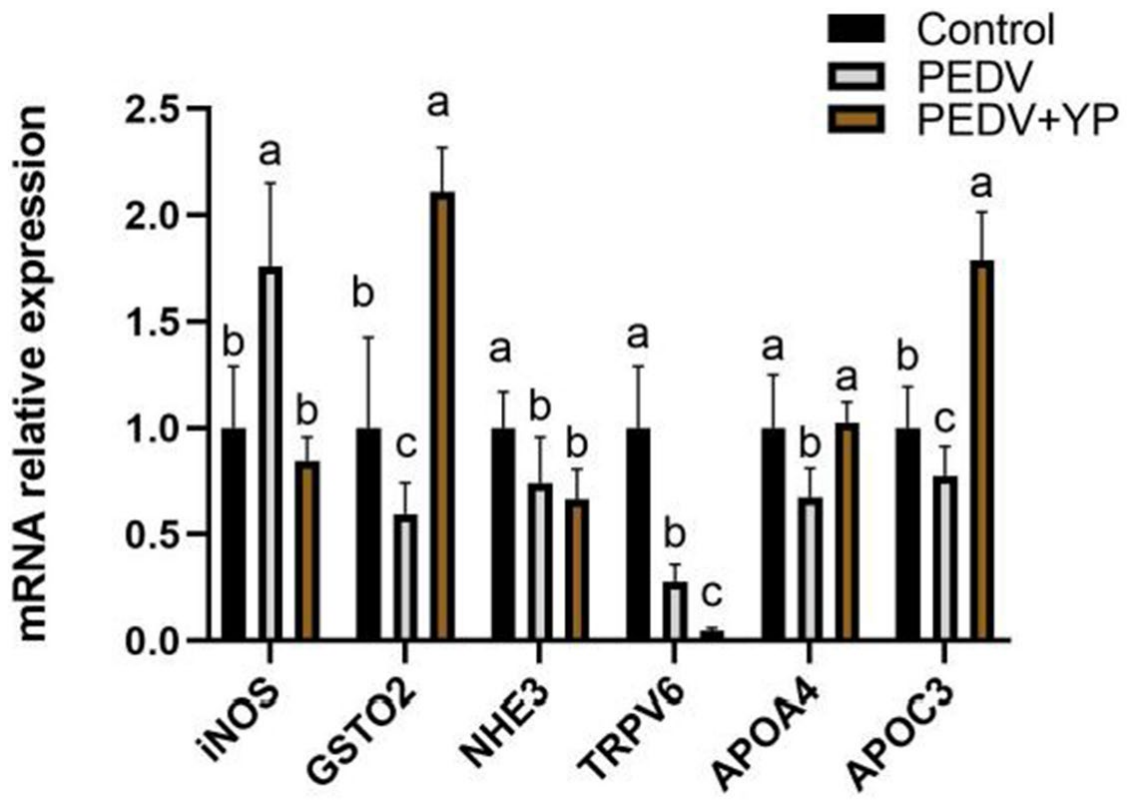
Effects of YP on the expression of mRNA expression levels of and detected by qPCR in the jejunum of piglets. Values are mean and pooled SEM, *n* = 6; a,b,c means with different superscripts differ, *p* < 0.05.

Data on iNOS, GSTO2, NHE3, TRPV5, APOA4 and APOC3 mRNA levels are summarized in Figure 5. Compared with control group, PEDV-infected piglets had a higher iNOS mRNA levels in the jejunum; lower GSTO2, NHE3, TRPV5, APOA4 and APOC3 mRNA levels in the jejunum (*p* < 0.05). Compared with PEDV group, piglets fed by YP had lower iNOS and TRPV6 mRNA levels in the jejunum; higher GSTO2, APOA4 and APOC3 mRNA levels in the jejunum (*p* < 0.05).

## 4 Discussion

The intestine is an organ for digestion and absorption and is also an important barrier between the host and the external environment (Qiu et al., 2020). Typical PED symptoms with multifocal to diffuse villus atrophy found in the present study were consistent with the previous studies (Lee et al., 2021). As an enterovirus, PEDV primarily infects enterocytes and causes severe damage in the small intestine (Lee, 2015). In addition, histopathological structures analysis directly reflected that YP alleviates intestinal lesions of piglets and improves the villi structures of piglets, similar to the previous study that yeast culture improves gut morphology of weanling pigs (van der Peet-Schwering et al., 2007; Fouhse et al., 2019). Intestinal Fatty Acid Binding Protein (iFABP) is a protein exclusively expressed by enterocytes. It is released into the circulation after enterocyte damage and has shown to be a good marker for the early identification of intestinal ischemia and damage (Relja et al., 2010; Schellekens et al., 2014). Amphiregulin (AREG), a member of the epidermal growth factor (EGF) family, has been shown to play an important role in wound healing and tissue repair (Zaiss et al., 2015). MMP13 is the main enzyme that targets cartilage degradation. MMP13 can not only degrade type II collagen in cartilage, but also proteoglycan, type IV and type IX collagen, osteonectin, and basement membrane proteoglycan (Vincenti and Brinckerhoff, 2002). In this study, PEDV infection resulted in increased mRNA levels of iFABP in the ileum. Zhang et al. Reported that PEDV infection increased the concentrations of intestinal fatty acid-binding protein (Zhang et al., 2019). Our results are consistent with it. Meanwhile, PEDV infection leads to increased the mRNA level of AREG in jejunum and ileum, possibly due to muscle atrophy caused by PEDV-induced malnutrition, this is consistent with the study of Hanata et al. (2021). The results indicate that PEDV can cause intestinal damage and affect the metabolic function of piglets. YP further enhances AREG, which may play a role in tissue repair. This study also showed that PEDV infection could increase the mRNA level of MMP13 in jejunum and ileum, and YP had no effect on it. It shows that PEDV affected the cartilage development of piglets, but YP had no effect on it. All in all, the present study suggests that YP could alleviate intestine injury and promote gut healing and tissue repair of piglets infected with PEDV.

TG is an indicator of lipid metabolism in animals (Hu et al., 2015). It is extremely essential for growing piglets except as energy storage (Farnworth and Kramer, 1987). It participates in various functions, including structure, signaling and thermal insulation (Bell et al., 1997). In this study, PEDV infection significantly decreased the content of TG in plasma, while YP significantly increased the content of TG. This is consistent with the study of Liu et al. (2015). Calcium plays a key role in many bio-homeostasis functions. Previous results showed that PEDV infection causes a significant difference in the intra- and extracellular Ca^2+^ concentrations, PEDV infection causes a significant increase in the flow of Ca^2+^ from the extracellular space into the cells (Kan et al., 2023). In this study, serum CA content in PEDV group decreased, which was consistent with the results of TRPV6 in subsequent experiments. YP adjusted the plasma CA content, but the mRNA level of TRPV6 was decreased in PEDV+YP group, indicating that the YP exerts the role by different pathways. Gamma-glutamyltransferase (GGT) enzyme activity is widely distributed in living organisms, including plants, yeasts and bacteria. GGT is able to hydrolyze the gamma-glutamyl bonds of glutathione (GSH) and glutathione S-conjugates (Corti et al., 2020), and can participate in maintaining the stability of intestinal redox function of piglets. Creatine kinase (CK) activity, through the creatine-kinase-phosphocreatine (CK/PCr) system, provides a temporal and spatial energy buffer to maintain cellular energetic homeostasis, being responsible to provide adenosine triphosphate (ATP) to the proper function of ATPases enzymes (Baldissera et al., 2018), Selsby et al.’s study (Selsby et al., 2015) showed that the increase of CK content in pig serum was caused by malnutrition. In this study, PEDV infection caused the increase of CK content. After piglets were given YP, CK content decreased. In brief, in our study, we found that YP can alleviate the nutrient loss and maintain the homeostasis in serum of piglets.

The capacity of the biosystem to detoxify the active intermediaries and balance the systematic phenomenon of reactive oxygen species can be reflected by oxidative stress (Cabello-Verrugio et al., 2016). Cells can protect themselves from oxygenates and other hydroxyl radicals by antioxidant enzymes (including GSH-Px, CAT and SOD) (Harris, 1992). MPO is a member of the superfamily of heme peroxidases that is mainly expressed in neutrophils and monocytes. Elevated MPO levels in circulation are associated with inflammation and increased oxidative stress (Ndrepepa, 2019), which is consistent with our findings, PEDV infection resulted in an increase in serum MPO activity, while YP significantly mitigated this increase. MDA as a marker is usually used to evaluate the level of oxidative stress (Del Rio et al., 2005). The main product of oxidative stress is H_2_O_2_ in the body (Sies, 2014). These two are classic oxidative metabolites. In this study, YP increased the activity of antioxidant enzymes in serum, ileum and colon, what’s more, it decreased the content of oxidation products in serum, duodenum and jejunum of PEDV infected piglets. Interestingly, SOD content in serum increased in PEDV group, which may be due to oxidative stress caused by PEDV and increased content of reactive oxygen species in serum, resulting in increased SOD and its ability to clear reactive oxygen species (Carillon et al., 2013). Our study shows that the supplementation of YP could alleviate oxidative stress induced by PEDV and improve anti-oxidative capacity. Moreover, GSTO2 perform a variety of vital functions, particularly in reducing oxidative damage (Zhang et al., 2016). In this study, from antioxidant results, YP reduced antioxidant damage, which was consistent with mRNA results. These results are consistent with existing studies (Sauerwein et al., 2007).

Among the structural proteins of PEDV, PEDV M protein is an essential structural protein implicated in viral infection, replication and assembly although the precise mechanisms underlying these functions remain enigmatic (Dong et al., 2021). PEDV N protein is the most abundant viral structural protein, which can be combined with viral genomic RNA to form ribonucleoprotein complexes, thereby participating in the transcription and replication of the virus (Zhai et al., 2023). The spike (S) protein plays pivotal roles in PEDV attachment, receptor binding, and virus–cell membrane fusion during PEDV invasion into host cells (Li et al., 2021). The PEDV S protein is also involved in the induction of neutralizing antibodies in the host (Chang et al., 2002). Studies have shown that YP has antimicrobial effects. M. Roselli et al. ‘s study showed that yeast extract protected the cells against the increased membrane permeability caused by *Escherichia coli* K88 (Roselli et al., 2007). Davis et al.’s study showed that β-glucan, the active compound of YP, is easily utilized by intestinal cells to effectively regulate the activity of macrophages and T lymphocytes (Davis et al., 2004), which was important to improving the resistance to virus infection. In this study, PEDV M, N,S mRNA levels in the YP group were significantly decreased, indicating that YP had a certain interference effect to PEDV colonization in jejunum and colon.

A low concentration of IL-1β mainly exerts an immunomodulatory effect, whereas a high concentration of IL-1β mainly stimulates the expression of inflammation and autoimmune disease-related genes, leading to fever and cachexia. Studies have shown that β-Glucans has good immune function (Liu et al., 2018). Inducible nitric oxide synthase (iNOS) is responsible for increased nitric oxide (NO) synthesis in tissues during inflammatory processes and premalign-malign transformation (Keklikoglu et al., 2008). It is known that during intestinal inflammation there is excessive NO production (Marion et al., 2003). iNOS expression and activity are also utilized to determine at which part of the intestinal tract the inflammatory processes occur (Lamarque et al., 2003). In this study, PEDV caused intestinal inflammation, YP mitigated this phenomenon, which is consistent with their research. REG3G, as a secreted protein, which has the functions of trophic, anti-inflammatory and anti-apoptosis (Zhang et al., 2023). IRF7 is a lymphoid-specific factor that is predominantly expressed in the cytoplasm of the spleen, thymus and peripheral blood lymphocytes, such as B cells, plasmacytoid dendritic cells (pDCs) and monocytes (Ma et al., 2023). Recent studies have revealed that IRF7 exerts a broad range of activities in different biological processes (Qing and Liu, 2023). In this study, YP significantly reduced the mRNA level of IL-1β in jejunum and ileum compared with PEDV group, this is consistent with the study of No et al. (2021). REG3G increases after PEDV infection because PEDV causes intestinal inflammation and triggers the body’s inflammatory response, this has been reported in the study of Sun et al. (2021). These results indicate that YP has a good anti-inflammatory effect. There is a study shows that many viruses have evolved to target IRF3 and IRF7 to inhibit or circumvent the activation of the two factors and promote viral replication (Li et al., 2021). This may be the reason for the decrease of IRF7 mRNA level in jejunum after PEDV infection in this study. However, YP has no callback effect on IRF7 expression. G.S. Jensen et al. ‘s study showed an anti-inflammatory effect of the XP yeast culture in conjunction with activation of NK cells and B lymphocytes *in vitro* (Jensen et al., 2008). Jianmin Zhou et al. ‘s study provided that dietary yeast cell wall polysaccharides alleviated the LPS-induced elevated levels of serum IL-6 and IL-1β and the up-regulated expression of IL-1β, TNF-α, IFN-γ, and IL-6 in spleen and/or ileal mucosa (Jianmin Zhou et al., 2023). Nevertheless, YP could relieve inflammation of the intestine in PEDV-infected piglets.

The jejunum is the longest part of the small intestine and is important for reflecting intestinal health. Therefore, we conducted further detection of jejunum related genes involved in metabolism and transport. The Na/H exchanger 3 (NHE3) mediates Na and fluid absorption in the intestine and reabsorption in the kidney (Xue et al., 2022). XUE et al. ‘s research shows that knockout of NHE3 selectively in the small intestine and colon of mice results in disruption of intestinal structural integrity, persistent alkaline diarrhea, metabolic acidosis, hyponatremia and hyperkalemia associated with drastically elevated plasma aldosterone levels, and increased mortality rate (Xue et al., 2022). This study showed that PEDV infection could lead to intestinal injury and diarrhea in piglets, while YP had no effect on NHE3 expression. The TRPV6 protein is expressed in a range of epithelial tissues such as the intestine, kidney, placenta, epididymis, and exocrine glands such as the pancreas, prostate and salivary, sweat and mammary glands. The TRPV6 gene is a direct transcriptional target of the active form of vitamin D and is efficiently regulated to meet the body’s need for Ca^2+^ demand (Khattar et al., 2022). This is consistent with the results of plasma biochemistry in our study. APOA4 is a lipoprotein primarily synthesized by enterocytes of the small intestine, and functions have been ascribed, which is involved in the metabolic procedure of lipid and glucose, and anti-inflammatory response (Fei Wang et al., 2015). Studies have shown that APOA4 knockout mice exhibited a significantly greater inflammatory response to DSS, which was reversed upon exogenous administration of APOA4 to knockout mice (Vowinkel et al., 2004). We found that PEDV induces intestinal inflammatory response, and YP plays an anti-inflammatory role, which has been reported by Bacha et al. (2017). Apolipoprotein C-III (APOC3) has a critical role in the metabolism of triglyceride (TG)-rich lipoproteins (TRLs). Animal models lacking the APOC3 gene exhibit reduced plasma TG levels, whereas the overexpression of APOC3 leads to increased TG levels (Norata et al., 2015). This is consistent with the results of plasma biochemical and mRNA levels in this study. This indicates that PEDV infection can lead to the decrease of blood lipids in piglets, and YP can increase blood lipids and reduce the damage of jejunum. The data proved that YP has the function of regulating small intestine metabolism. PEDV infection caused a mild inflammatory response in the colon without causing intestinal tissue damage, which may be due to the fact that the colon belongs to the large intestine and only has water reabsorption and nutrient digestion and absorption do not account for much (Zhang et al., 2018). In addition, the YP positive control group was not set in this study, to make the results more concise, because the present study aimed to investigate the intervention effect of YP on PEDV infection. However, more research will be conducted in the future, to comprehensively explore the effect of YP on regulating small intestine function, such as setting another positive control group fed only with YP.

## 5 Conclusion

In summary, PEDV causes intestinal injury, intestinal oxidative stress, inflammatory response, and metabolism disorder in piglets. YP could inhibit virus replicates, improve intestinal morphology, enhance antioxidant capacity, relieve inflammation and regulate the metabolism of the intestine in PEDV-infected piglets.

## Data availability statement

The original contributions presented in the study are included in the article/supplementary material, further inquiries can be directed to the corresponding author.

## Ethics statement

The animal study was approved by the Institutional Animal Care and Use Committee of Wuhan Polytechnic University (Number: 202209004). The study was conducted in accordance with the local legislation and institutional requirements.

## Author contributions

HL: Data curation, Writing – original draft, Writing – review & editing, Project administration, Validation. MW: Data curation, Software, Supervision, Writing – review & editing. ZL: Methodology, Project administration, Writing – review & editing. QZ: Investigation, Methodology, Writing – review & editing. XZ: Project administration, Writing – review & editing. YZ: Methodology, Project administration, Supervision, Writing – review & editing. DZ: Methodology, Software, Writing – review & editing. LW: Data curation, Methodology, Supervision, Writing – review & editing. YH: Conceptualization, Funding acquisition, Methodology, Project administration, Resources, Writing – review & editing. TW: Conceptualization, Funding acquisition, Investigation, Methodology, Project administration, Resources, Writing – review & editing.
